# PK/PD studies on non-selective PDE inhibitors in rats using cAMP as a marker of pharmacological response

**DOI:** 10.1007/s00210-017-1406-z

**Published:** 2017-07-20

**Authors:** Artur Świerczek, Elżbieta Wyska, Sebastian Baś, Marta Woyciechowska, Jacek Mlynarski

**Affiliations:** 1 Department of Pharmacokinetics and Physical Pharmacy, Jagiellonian University, Medical College, 9 Medyczna Street, 30-688 Kraków, Poland; 2 Faculty of Chemistry, Jagiellonian University, Ingardena 3, 30-060 Kraków, Poland

**Keywords:** cAMP, PK/PD modelling, PDE inhibitors, Inflammation, Biomarker

## Abstract

In recent years, phosphodiesterase (PDE) inhibitors have been frequently tested for the treatment of experimental inflammatory and immune disorders. It is suggested that anti-inflammatory properties of PDE inhibitors are related to their ability to increase cAMP levels. The aim of this study was to verify the hypothesis that cAMP may be a useful marker of pharmacological response following administration of non-selective PDE inhibitors (pentoxifylline and (±)-lisofylline) to endotoxemic rats. Male Wistar rats were administered LPS (1 mg kg^−1^, i.v.) simultaneously with either compound given at two doses (40 and 80 mg kg^−1^, i.v.). Levels of cAMP and both compounds in animal plasma were measured by the validated HPLC methods. Pharmacokinetic-pharmacodynamic analysis was performed using basic and modified indirect response (IDR) models II in Phoenix WinNonlin. The results of this study indicate that, in contrast to pentoxifylline, (±)-lisofylline demonstrates a non-linear pharmacokinetics in rats with endotoxemia. In vitro study using human recombinant PDE4B and PDE7A revealed the occurrence of additive interaction between studied compounds. Moreover, (±)-lisofylline is a more potent inhibitor of PDEs compared to pentoxifylline, as evidenced by lower IC_50_ values. Following administration of both compounds, levels of cAMP in rat plasma increased in a dose-dependent manner. The modified IDR model II better described cAMP levels over time profiles. The validity of the proposed marker was confirmed by measuring plasma TNF-α levels in the studied animals. In conclusion, cAMP may be used in future preclinical and clinical studies of some PDE inhibitors to evaluate the drug concentration–effect relationship.

## Introduction

In recent years, phosphodiesterase (PDE) inhibitors have been frequently tested as a potential treatment in sepsis and some autoimmune disorders (Harada et al. 1989; Yang et al. 2005; González-García et al. 2013). Especially, selective PDE4, PDE7, or dual PDE4/7 inhibitors are widely studied as immunomodulatory agents (Jankowska et al. 2017). Progression of sepsis and autoimmune diseases depends on various factors, but in both cases, the balance between T helper 1 (Th1) and T helper 2 (Th2) lymphocytes has been indicated as the most important one (Ferguson et al. 1999; Kunz and Ibrahim 2009). Th1 lymphocytes produce pro-inflammatory cytokines such as interferon-γ (INF-γ), interleukin-2 (IL-2) or tumour necrosis factor α (TNF-α), while Th2 lymphocytes release anti-inflammatory cytokines, such as IL-4 and IL-5. Depending on which cytokines predominate in plasma and target tissues, progression or remission of the disease is observed (Selmi 2011; Noack and Miossec 2014; László et al. 2015). The current treatment of sepsis and autoimmune diseases is not always efficient and often associated with severe side effects; therefore, a search for new anti-inflammatory drugs is needed in order to improve the therapeutic outcome in these diseases.

One of the most important steps in research and development of new drugs is to find a suitable marker of pharmacological response. In experimental animal models of immune disorders, cytokines such as interleukins (e.g. IL-6, IL-10) and TNF-α or nitric oxide (NO) are usually used for this purpose (Gozzi et al. 1999; Chakraborty et al. 2005; Wyska 2010a; Lon et al. 2012). However, these biomarkers have some limitations. First of all, their levels measured in different species and in different individuals of the same species differ significantly. Moreover, the increase in their concentrations is observed with some delay relative to the stimulus (Lon et al. 2012). For these reasons, there is a need to search for new biomarkers that are closely correlated with the observed pharmacological effect in a dose-dependent manner.

PDEs are responsible for 3′,5′-cyclic adenosine monophosphate (cAMP) and 3′,5′-cyclic guanosine monophosphate (cGMP) degradation; thus, the main mechanism of action of PDE inhibitors is related to an increase in levels of these nucleotides. Some PDE families are cGMP-specific (PDE5, 6 and 9), some hydrolyse both cAMP and cGMP (PDE1, 2, 3, 10 and 11) and others are cAMP-specific (PDE4, 7 and 8). Due to ubiquitous expression of PDE4 in mammalian organisms, this enzyme is primarily responsible for the cAMP degradation in the human body (Houslay et al. 2005; Francis et al. 2011). The clinical impact of changes in cAMP levels remains not entirely clarified, but nevertheless, it has been shown that an increase in cAMP amounts has an immune-weakening potential. On the other hand, the reduction of cAMP levels has an immunostimulatory effect (Raker et al. 2016).

Pentoxifylline (PTX) and lisofylline (LSF) are non-selective PDE inhibitors that undergo metabolic interconversion (Wyska et al. 2006). Thus, irrespective of which one is administered, both compounds are present in blood. For many years, they have been widely used in animal studies on inflammation and autoimmune disorders (Rice et al. 1994; Bright et al. 1998; Yang et al. 2002, 2003; Wyska 2010a). PTX is a drug commonly used in the treatment of intermittent claudication. Moreover, it has been tested clinically in the treatment of diseases, such as sepsis or pulmonary sarcoidosis in humans (NCT02163174, NCT00001877). R-(-)-LSF is an enantiomer of PTX metabolite M1 recently investigated as a potential treatment of autoimmune diabetes (NCT01603121).

It is well known that PDE inhibitors exert their anti-inflammatory activity, at least in part, by increasing concentrations of cAMP. Therefore, it seems that this nucleotide may serve as a marker of drug response in experimental models of inflammatory disorders. To confirm this assumption, we evaluated the influence of two non-selective PDE inhibitors: PTX and (±)-LSF on cAMP levels in plasma of rats with lipopolysaccharide (LPS)-induced endotoxemia. Based on these data, two pharmacokinetic/pharmacodynamic (PK/PD) models were developed to describe the relationship between plasma concentrations of both compounds and their active metabolites and pharmacological response. The potencies of PTX and both enantiomers of LSF as inhibitors of human recombinant PDE4B (hrPDE4B) and PDE7A (hrPDE7A) were preliminarily assessed using an in vitro assay. To select an appropriate PK/PD model, the nature of interaction between PTX and R-(-)-LSF or PTX and (±)-LSF was evaluated in vitro using human recombinant PDE4B enzyme by the combination index (CI) analysis. The validity of cAMP as a biomarker was verified by measuring TNF-α levels in plasma of endotoxemic rats treated with the studied compounds.

## Materials and methods

### Reagents

cAMP sodium salt, PTX (1-(5-oxohexyl)-3,7-dimethylpurine-2,6-dione), dimethyl sulfoxide (DMSO), LPS (*Escherichia coli* 055:B5) and 50% 2-chloroacetaldehyde water solution were purchased from Sigma-Aldrich (Germany). Temazepam (7-chloro-3-hydroxy-1-methyl-5-phenyl-3H-1,4-benzodiazepin-2-one) was a gift from Polfa (Poland). IBMX (1-methyl-3-(2-methylpropyl)-7H–purine-2,6-dione) was purchased from Santa Cruz Biotechnology (USA). (±)-LSF ((±)-1-(5-hydroxyhexyl)-3,7-dimethylpurine-2,6-dione) was obtained from the Department of Organic Chemistry, Faculty of Chemistry, Jagiellonian University (Cracow, Poland). R-(-)-LSF and S-(+)-LSF were isolated by chromatographic separation of racemate using a chiral semi-preparative Chiralpak AD column (Daicel Corp., Japan). Other chemicals were of high-performance liquid chromatography or analytical reagent grade and were purchased from Merck (Germany).

### In vitro PDE assay

The PDE inhibitory activity of PTX, R-(-)-LSF and S-(+)-LSF was evaluated using the PDE-Glo Phosphodiesterase Assay according to the manufacturer’s instruction (Promega Corporation, Madison, WI, USA). Briefly, 1,5 μl of 1× PDE-Glo reaction buffer containing 10 mU of purified hrPDE4B or hrPDE7A (SignalChem, Richmond, Canada) was pipetted into 384-well plate wells (Thermo Scientific, USA). The tested compounds were dissolved in DMSO, and a serial dilution of the inhibitors was performed using 1× PDE-Glo reaction buffer. Then, 1 μL of diluted inhibitors and 2.5 μL of cAMP solution were added to each well. After 10 min of incubation in 30 °C, 2.5 μL of PDE-Glo™ Termination Buffer and 2.5 μL of PDE-Glo™ Detection Solution were added and the plate was incubated for 20 min at room temperature. Finally, 10 μL of Kinase-Glo® Reagent was pipetted to each well and after 10 min of incubation, the luminescence was measured using a microplate luminometer (POLARstar Omega, BMG LABTECH, Ortenberg, Germany). All data points are the average of two determinations.

### Combination index analysis

CompuSyn (ComboSyn, Inc., Paramus, NJ, USA) computer program was used in the calculation of the CI of non-constant ratio combinations of PTX with R-(-)-LSF or PTX with (±)-LSF as hrPDE4B inhibitors. The CI was evaluated based on the Combination Index-Isobologram Theorem (Chou 2006). CI values = 1, >1 and <1 indicate an additive effect, antagonism and synergism, respectively. However, values between 0.9 and 1.1 are considered as nearly additive. To this end, four concentrations of each drug producing between 25 and 75% of the maximal inhibitory potency (data obtained from the single compound study) were chosen. In the next step, four concentrations of PTX and R-(-)-LSF or PTX and (±)-LSF were mixed with each other to produce 32 combinations of investigated compounds (16 combinations for each pair of compounds). The inhibitory potencies of all combinations were measured using the described above PDE-Glo Phosphodiesterase Assay.

### Animals

Male Wistar rats weighting 250–300 g were housed in conditions of the constant temperature with a 12:12 h light–dark cycle with free access to food and water. The animals were implanted with catheters (SAI Infusion Technologies, USA) in the jugular vein under ketamine/xylazine anaesthesia 2 days prior to the experiment. Before drug administration, the rats were fasted overnight with free access to water. All animal procedures were approved by the First Local Ethical Committee on Animal Testing at the Jagiellonian University. All applicable international, national and/or institutional guidelines for the care and use of animals were followed.

### In vivo experimental design

All compounds were directly dissolved in 0.9% sterile saline and used within 1 day of preparation. The animals were divided into five groups (*n* = 3–4). The control group received LPS alone at a dose of 1 mg kg^−1^, whereas the other four groups received one of the tested compounds (PTX or (±)-LSF) at a dose of 40 or 80 mg kg^−1^ simultaneously with LPS given at a dose of 1 mg kg^−1^. All injections (1 mL kg^−1^) were given to the tail vein of the rat under isoflurane anaesthesia. Blood samples were collected from the jugular vein catheter into snap-cap propylene tubes containing heparin at 0, 15, 30, 60, 75 and 90 min following administration of PTX and at 0, 15, 30, 60, 90 and 120 min following (±)-LSF dosing. Subsequently, samples were kept on ice and centrifuged for 10 min at 3000×*g* at 4 °C (EBA 12 R, Hettich, Germany). The individual plasma samples were harvested and stored at −80 °C until analysis.

### Analytical methods

In order to isolate studied compounds and their metabolites from plasma, to 100 μL of plasma (or plasma spiked with methanol standard solution of PTX or (±)-LSF), 10 μL of temazepam (internal standard) solution in methanol and 20 μL of 1 M hydrochloric acid water solution were added. Then, the samples were extracted with 3 mL of dichloromethane for 20 min on a shaker (VXR Vibrax, IKA, Germany). Subsequently, all tubes were centrifuged (2000×*g*, 15 min) and organic layers were transferred to new glass tubes and evaporated under gentle stream of nitrogen at 37 °C. Dry residues were dissolved in 100 μL of mobile phase and placed in the autosampler vials. The volume of injection was set to 50 μL. The HPLC system (LaChrom Elite, Merck-Hitachi, Darmstadt, Germany) consisted of an L-2130 pump, an L-2200 autosampler, an L-2450 diode array detector and an L-2350 column oven. EZChrome Elite v. 3.2 (Merck-Hitachi) software was used for data acquisition. The analysis was performed on a LiChrospher 100 RP-18 column (250 mm × 4 mm) with a particle size of 5 μm protected with a LiChroCART (4 mm × 4 mm) guard column (Merck, Germany). The mobile phase consisted of dioxan, acetonitrile and aqueous solution of acetic acid (pH = 3.0) mixed at the ratio of 6.5:6.5:87 (*v*/*v*/*v*), respectively, and pumped at a flow rate of 1.2 mL min^−1^. Analytical wavelength was set to 275 nm and temperature of separation was 35 °C. In these conditions, the retention times were found to be 15.5 min for temazepam, 17.5 min for PTX and 21 min for (±)-LSF.

The levels of cAMP in rat plasma were measured using the same HPLC system and a fluorescence detector (model FL-2485). The analysis was performed after incubation of plasma with 2-chloroacetaldehyde as a derivatization reagent in elevated temperature to form the fluorescing derivative—1,N^6^-etheno-cAMP. To 100 μL of plasma (or plasma spiked with water standard solution of cAMP), 15 μL of 2 M 2-chloroacetaldehyde solution and 50 μL of 0.5 M acetate buffer (pH = 4.5) were added to a snap-cap tubes. Samples were then vortex-mixed (Reax top, Heidolph, Germany) for 20 s and incubated at 80 °C for 20 min. Thereafter, the reaction mixture was cooled on ice, the samples were centrifuged (3000×*g*, 10 min) and 100 μL of supernatants was placed in the autosampler vials. The volume of injection was 50 μL. The separation was performed at isocratic conditions at 35 °C using the mobile phase composed of 20 mM citric-phosphate buffer (pH = 3.2) and methanol mixed at the ratio of 91:9 (*v*/*v*) and pumped at a flow rate of 1.3 mL min^−1^. The excitation and emission wavelengths were set to 280 and 420 nm, respectively. In these conditions, the retention time of 1,N^6^-etheno-cAMP was 6 min.

No interfering peaks were observed at the retention times of the analytes and the internal standard. The calibration curves for (±)-LSF and PTX constructed by plotting the peak area ratios of the analytes to the internal standard versus corresponding concentrations of analytes were linear in a range of 0.1 to 125 μg mL^−1^. For cAMP, the calibration curve was obtained by plotting the peak areas of cAMP versus corresponding concentrations of this nucleotide and it was linear in a range of 10 to 500 pmol mL^−1^. All values for accuracy and precision were within the recommended limits (EMA 2011).

Plasma TNF-α levels were measured by rat TNF-α Quantikine ELISA kit (R&D Systems, Minneapolis, MN, USA) according to the manufacturer’s protocol. The limit of quantification was 12.5 pg mL^−1^.

### Pharmacokinetics

Two independent PK models were developed for each parent drug, namely PTX or (±)-LSF, and their active metabolites following intravenous (i.v.) route of administration. To this end, one- or two-compartment pharmacokinetic models with linear or Michaelis–Menten type saturable elimination from the central compartment were tested. Both dose levels in each case were simultaneously fitted to obtain a single set of parameters. The final pharmacokinetic models for PTX and (±)-LSF were selected on the basis of visual inspection of the fitting, examination of residuals, parameter precision, Akaike Information Criteria (AIC), Bayesian Information Criteria (BIC) and analysis of the correlation matrix. Pharmacokinetic analysis was performed using Phoenix WinNonlin v. 6.3 (Pharsight Corp, Certara*,* St. Louis, MO, USA).

### Pharmacodynamics

**Fig. 1:**
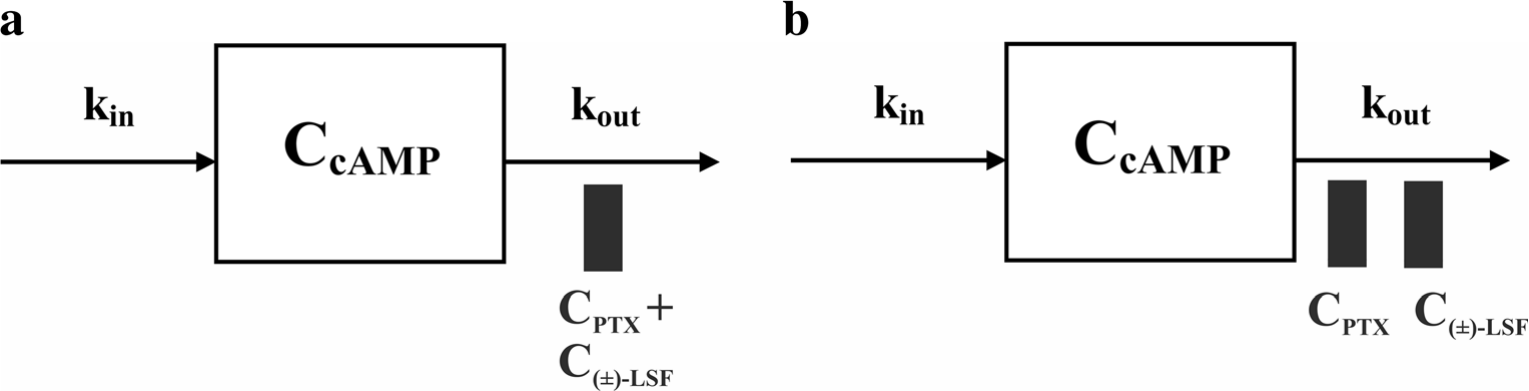
Schematic representations of the proposed pharmacodynamic models following intravenous administration of PTX or (±)-LSF to endotoxemic rats: the simple IDR model II (**a**) and the additive interaction IDR model II (**b**)

The indirect response (IDR) model II was employed to describe pharmacodynamics of PTX and (±)-LSF as non-selective PDE inhibitors (Fig. 1). This selection was based on the fact that both compounds inhibit degradation of cAMP used in this study as a marker of drug response.

Changes in plasma cAMP concentrations (C_cAMP_) over time following each compound administration may be described by the following equation:

1 $$ \frac{dC_{cAMP}}{dt}={k}_{in}-{k}_{out}\cdot I(t)\cdot {C}_{cAMP} $$

where *k*_*in*_ is zero-order cAMP production rate constant and *k*_*out*_ is the first-order cAMP elimination rate constant. In the absence of drug, the response stays at the baseline value (R_0_):

2 $$ {R}_0=\frac{k_{in}}{k_{out}} $$

Thus, k_in_ can be calculated as *R*_0_ k_out_, where *R*_0_ is the mean cAMP concentration measured before drug administration. The joint effect of PTX and (±)-LSF as parent drugs and metabolites was modelled using two approaches.

The first approach (Fig. 1a) was based on the assumption that both compounds, PTX and (±)-LSF, act with similar potencies; thus, inhibitory function may be described by the following equation:

3 $$ I(t)=1-\frac{I_{\max}\cdot {C_p}^{\gamma }}{{IC_{50}}^{\gamma }+{C_p}^{\gamma }} $$

where *C*_*p*_ is a sum of PTX and (±)-LSF plasma concentrations, *I*_max_ is the maximum ability of both compounds to inhibit k_out_, *IC*_50_ is plasma concentration of the combination of parent compound and metabolite at which inhibition is half-maximal, and *γ* is the Hill coefficient.

The second approach assumes that both compounds under investigation act with different potencies. Based on the results of the in vitro study, an additive interaction model was used (Eq. 4):

4 $$ I(t)=\left(1-\frac{I_{\max, PTX}\cdot {C_{PTX}}^{\gamma_{PTX}}}{{IC_{50,PTX}}^{\gamma_{PTX}}+{C_{PTX}}^{\gamma_{PTX}}}\right)\cdot \left(1-\frac{I_{\max, LSF}\cdot {C_{LSF}}^{\gamma_{LSF}}}{{IC_{50,LSF}}^{\gamma_{LSF}}+{C_{LSF}}^{\gamma_{LSF}}}\right) $$

where *I*_max,*PTX*_ and *I*_max,LSF_, are the maximum ability of PTX and (±)-LSF, respectively, to inhibit *k*_*out*_; *IC*_50,PTX_ and *IC*_50,LSF_ are the concentrations of PTX and (±)-LSF producing 50% of the maximum inhibition; *C*_*PTX*_ and *C*_*LSF*_ are plasma concentrations of PTX and (±)-LSF and γ_PTX_ and γ_LSF_ are the Hill coefficients. *I*_max_ and *γ* in Eqs. (3) and (4) were fixed to 1 during fitting procedure. To obtain a single set of pharmacodynamic parameters for each model, cAMP plasma concentrations following administration of PTX or (±)-LSF at both dose levels (40 or 80 mg kg^−1^) were fitted simultaneously.

### Statistical analysis

The peak cAMP and TNF-α plasma levels following administration of LPS alone or LPS simultaneously with both doses of each compound were compared using a one-way ANOVA and Tukey’s HSD post hoc test. The normality of data distribution was checked by Shapiro*–*Wilk test. The relationship between decimal logarithms of peak cAMP and TNF-α plasma concentrations was verified by least squares linear regression analysis. All statistical analyses were performed using Statistica v. 12 (StatSoft Inc., USA). The significance level *p* was set at 0.05.

## Results

### PDE inhibitory activity and CI analysis

**Fig. 2:**
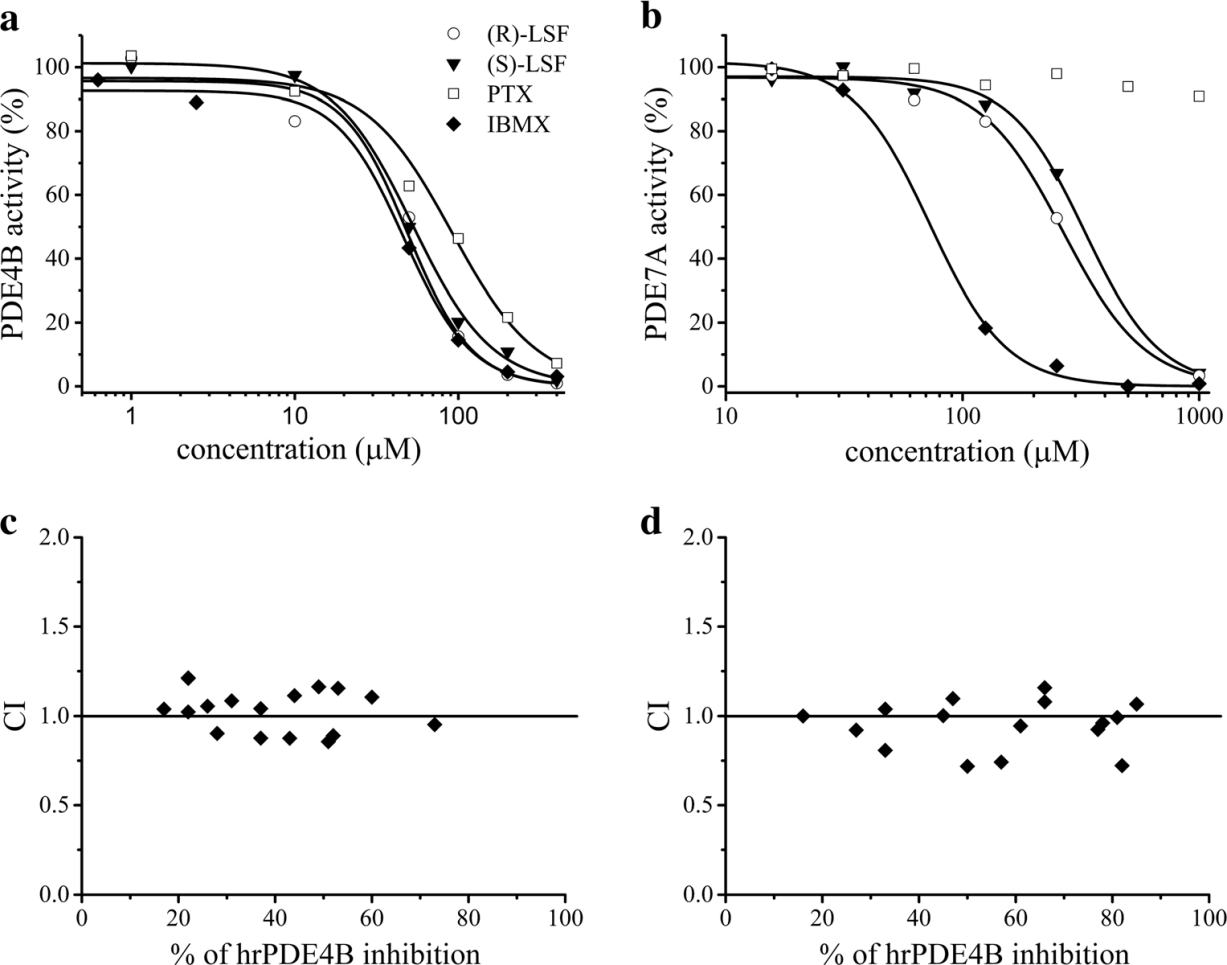
Observed (*symbols*) vs. predicted (*lines*) hrPDE4B (**a**) or hrPDE7A (**b**) activity (%) in the presence of the investigated compounds at different concentrations; CI plots of 16 combinations of PTX and R-(-)-LSF (**c**) or PTX and (±)-LSF (**d**) as hrPDE4B inhibitors

As presented in Fig. 2a, b, and in Table 1, both enantiomers of (±)-LSF inhibited hrPDE4B in the in vitro assay with similar potencies. PTX in the same test acted slightly weaker. R-(-)-LSF and S-(+)-LSF act also as weak inhibitors of hrPDE7A, whereas PTX as an inhibitor of this enzyme was ineffective. IBMX, a non-selective PDE inhibitor used as a reference compound in this study, reached PDE7A IC_50_ value about 3 and 4 times lower than those of R-(-)-LSF and S-(+)-LSF, respectively. Moreover, this compound acted two times stronger than PTX and similarly to both enantiomers of LSF as a hrPDE4B inhibitor.

**Table 1:** IC_50_ values of studied inhibitors estimated by non-linear regression using data obtained from in vitro study

Compound	PDE4B IC_50_ (μM)	PDE7A IC_50_ (μM)
R-(-)-LSF	49.1	266.1
S-(+)-LSF	52.5	328.2
PTX	91.3	>500.0
IBMX	46.6	77.7

CI values of each compound combination were plotted on CI plots, where the effect value (x axis) is a summary % of hrPDE4B inhibition of each combination (Fig. 2c, d). CI values obtained for seven of the 16 combinations of PTX and R-(-)-LSF (Fig. 2c) and for 11 of 16 combinations of PTX and (±)-LSF (Fig. 2d) were in the range of 0.9–1.1, indicating the existence of simple additive interaction. CI calculated for other combinations slightly exceeded the range of 0.9–1.1 indicating the presence of weak antagonism or synergism. However, the mean CI value obtained for all combinations of PTX and R-(-)-LSF was 1.02 (±0.12), and for PTX and (±)-LSF combinations, it was 0.95 (±0.14).

### Pharmacokinetics

**Fig. 3:**
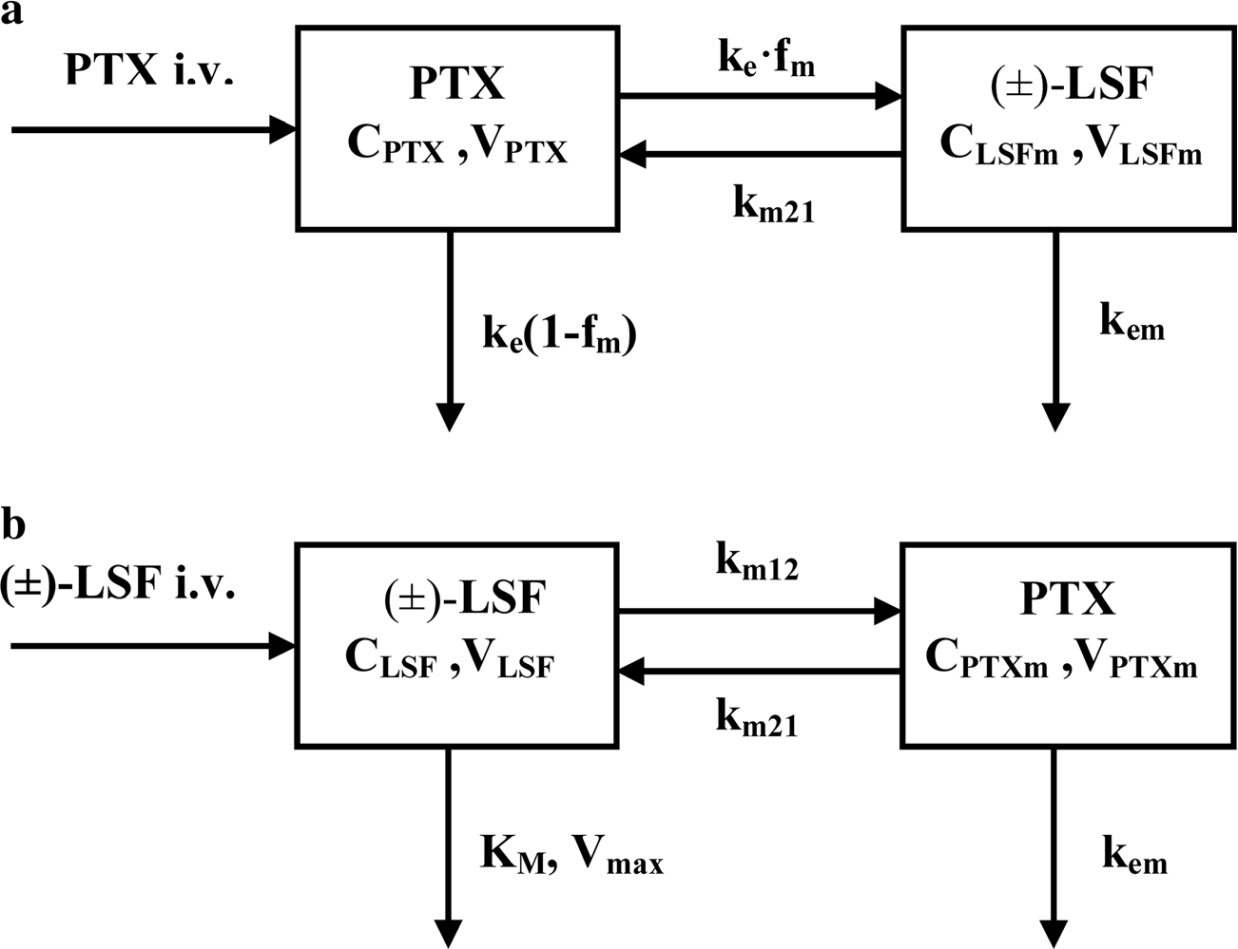
Schematic representations of the proposed pharmacokinetic models of PTX (**a**) and (±)-LSF (**b**) following intravenous administration of each compound to rats; C_**PTX**_ and C_**LSF**_, plasma concentrations of PTX and (±)-LSF; V_**PTX**_ and V_**LSF**_, volumes of PTX and (±)-LSF compartments, respectively; V_PTXm_ and V_LSFm_, volumes of PTX and (±)-LSF metabolite compartments, respectively; k_m12_, first-order conversion rate constant of parent compound into metabolite; k_m21_, first-order conversion rate constant of metabolite into parent compound; k_e_, first-order rate constant for disappearance of parent compound; f_m_, fraction of parent compound metabolized; k_em_, first-order elimination rate constant of metabolite; V_max_, maximal elimination rate constant; K_m_, drug concentration at which the elimination rate is half-maximal

Pharmacokinetic analysis revealed that a one-compartment model with linear elimination from the central compartment (Fig. 3a) best described PTX and (±)-LSF (as metabolite) concentration versus time profiles after i.v. administration of PTX to rats. In turn, a one-compartment model with Michaelis-Menten type elimination from the central compartment (Fig. 3b) was most appropriate to fit the plasma concentration versus time data of (±)-LSF and PTX (as metabolite) after (±)-LSF i.v. administration to rats. In both models, a mutual interconversion of the parent compounds and their metabolites was taken into account.

**Fig. 4:**
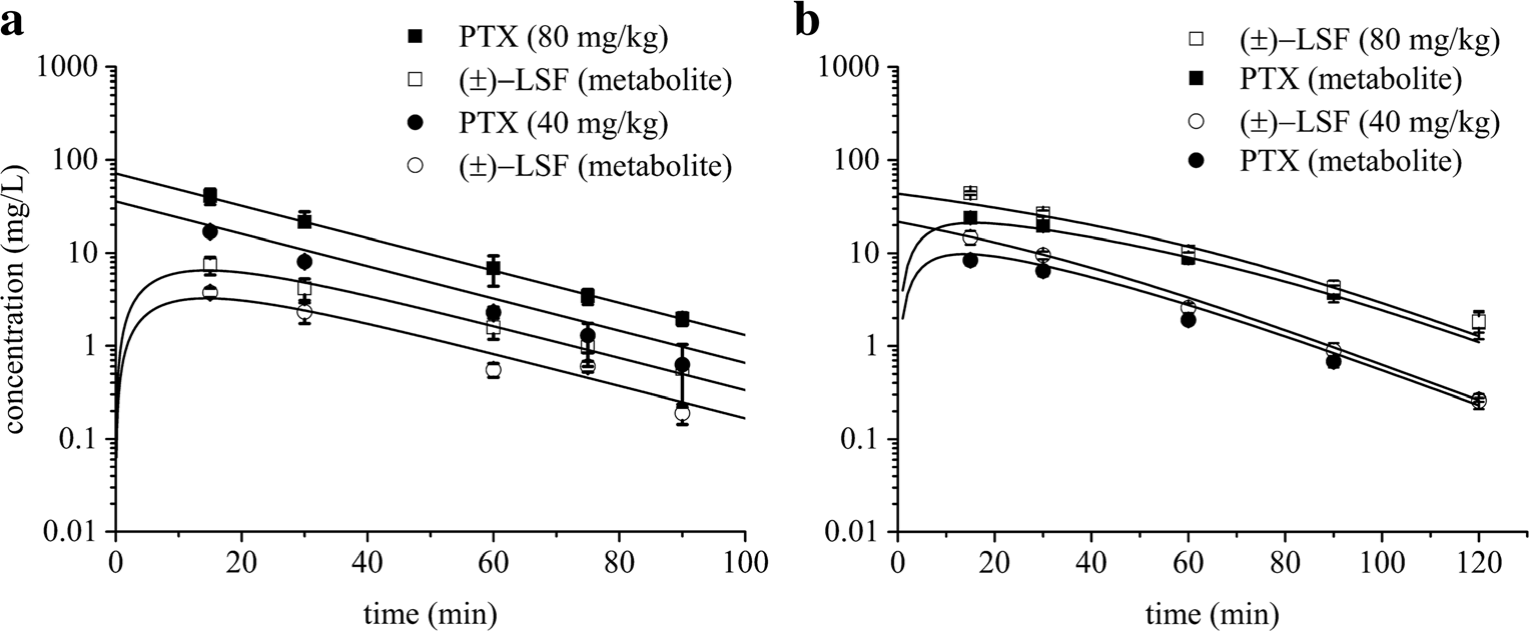
Mean (±SD) observed (*symbols*) and model predicted (*lines*) PTX and (±)-LSF plasma concentrations after PTX (**a**) or (±)-LSF (**b**) administration at two doses (40 or 80 mg kg^−1^) to rats (*n* = 3–4)

As presented in Fig. 4, the proposed models very well captured the concentration versus time data of both parent compounds and their respective metabolites.

The estimates of pharmacokinetic parameters are listed in Table 2.

**Table 2:** Estimated values of pharmacokinetic parameters of PTX or (±)-LSF as parent compounds

(±)-LSF (parent drug)		PTX (parent drug)	
Parameter	Final estimate (CV%)	Parameter	Final estimate (CV%)
V_LSF_ (L kg^−1^)	1.22 (3.44)	V_PTX_ (L kg^−1^)	1.07 (1.24)
V_PTXm_ (L kg^−1^)	2.01 (9.48)	V_LSFm_/f_m_ (L kg^−1^)	1.77 (15.23)
V_max_ (mg min^−1^ kg^−1^)	0.433 (56.85)	k_e_ (min^−1^)	0.056 (3.04)
K_m_ (mg L^−1^)	6.02 (91.82)	k_m21_ (min^−1^)	0.015 (9.89)
k_m12_ (min^−1^)	0.203 (12.74)	k_em_ (min^−1^)	0.157 (15.34)
k_m21_ (min^−1^)	0.095 (12.87)		
k_em_ (min^−1^)	0.116 (9.75)		

### Pharmacodynamics

The administration of investigated compounds to rats caused an inhibition of PDEs and a subsequent increase in cAMP levels in rat plasma in a dose-dependent manner with the peak concentration attained between 30 and 60 min post-(±)-LSF or PTX dosing. The first tested PK/PD model (Fig. 1a) is a basic IDR model II, where C_p_ is a sum of the parent drug and its active metabolite concentrations. The second tested model (Fig. 1b) is an additive interaction IDR model II, where the occurrence of a simple additive interaction between studied compounds is assumed. Both models describing endogenous cAMP levels consisted of one zero-order input (k_in_) and one first-order cAMP elimination rate constant (k_out_).

**Fig. 5:**
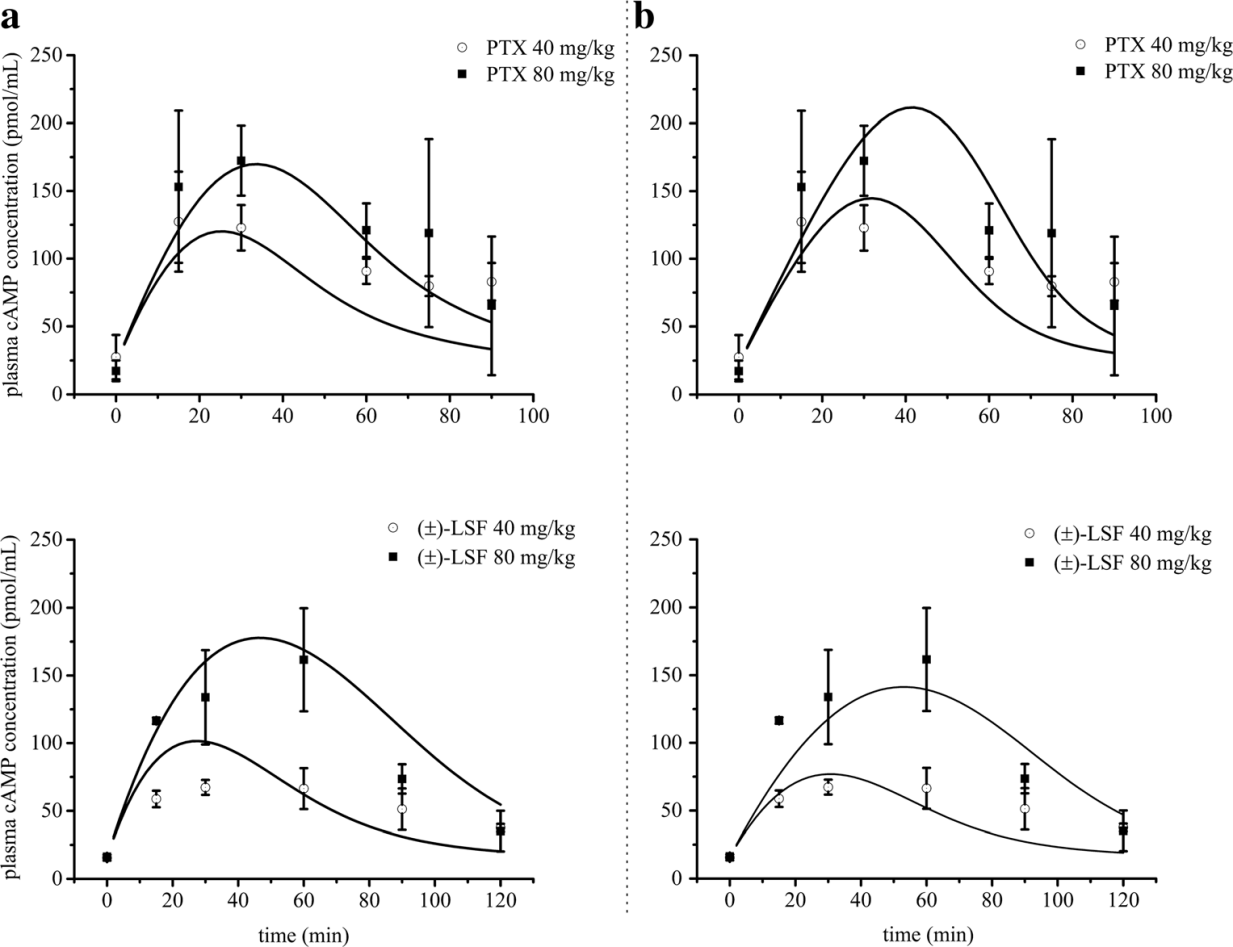
Mean (±SD) observed (*symbols*) and predicted (*lines*) based on the basic IDR model II (**a**) and additive interaction IDR model II (**b**) plasma cAMP concentrations after PTX or (±)-LSF administration at two doses (40 or 80 mg kg^−1^) to rats (*n* = 3–4)

The appropriateness of these models was evaluated on the basis of goodness-of-fit criteria*.* The second model (Fig. 1b) was found to better characterize changes in cAMP levels over time in the presence of both PDE inhibitors. An improvement in the fitting was observed, when comparing to the first tested model, as can be determined by visual inspection of the fitting (Fig. 5). Moreover, the AIC and BIC values for the interaction model (229.2 and 232.7, respectively) were lower in comparison with the values estimated for basic IDR model II (232.9 and 235.3, respectively). Pharmacodynamic parameters estimated using both models are listed in Table 3.

**Table 3:** Pharmacodynamic parameters estimated using both tested pharmacodynamic models following intravenous administration of PTX or (±)-LSF to rats challenged with LPS

	Basic indirect response model II	Interaction indirect response model II
Parameter	Final estimate (CV%)	Final estimate (CV%)
k_in_ (pmol mL^−1^ min^−1^)	9.709 (NE^a^)	6.650 (NE^a^)
k_out_ (min^−1^)	0.511 (14.06)	0.350 (10.54)
IC_50, PTX_ (mg L^−1^)	–	4.478 (22.24)
IC_50, LSF_ (mg L^−1^)	–	3.428 (19.07)
IC_50_ (mg L^−1^)	2.243 (9.28)	–

^**a**^Not estimated

A slightly lower value of IC_50_ estimated using the interaction model for (±)-LSF suggests that this compound may be more potent than PTX as an inhibitor of PDEs in vivo.

**Fig. 6:**
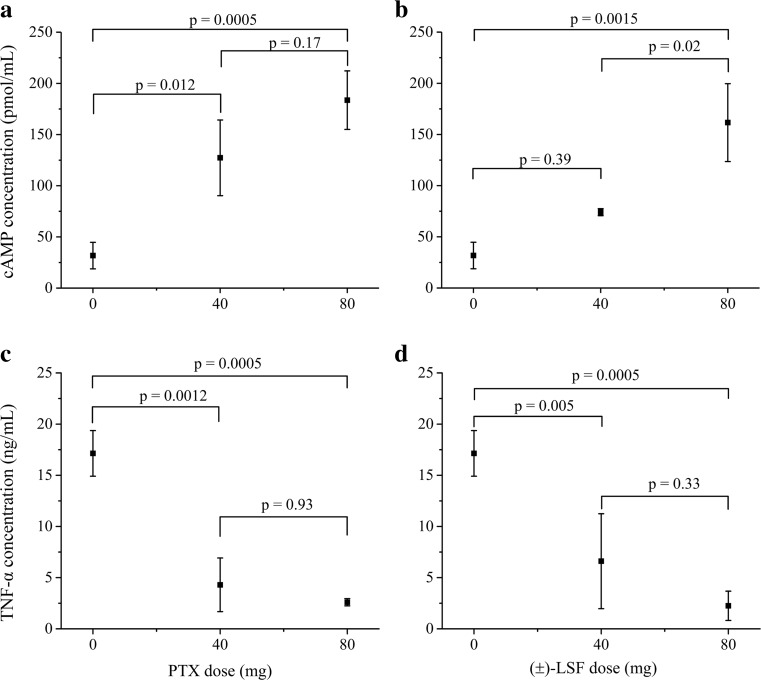
Effects of different doses of PTX and (±)-LSF on the peak cAMP plasma concentrations (±SD) (*n* = 4) (**a**, **b**) and on TNF-α plasma concentrations (±SD) (**c**, **d**) at 90 min following i.v. administration of each compound concomitantly with LPS (*n* = 4)

To verify whether cAMP levels increased in a dose-dependent manner, the peak cAMP plasma concentrations following administration of LPS only and LPS simultaneously with two different doses of either compound were compared statistically (Fig. 6a, b).

**Fig. 7:**
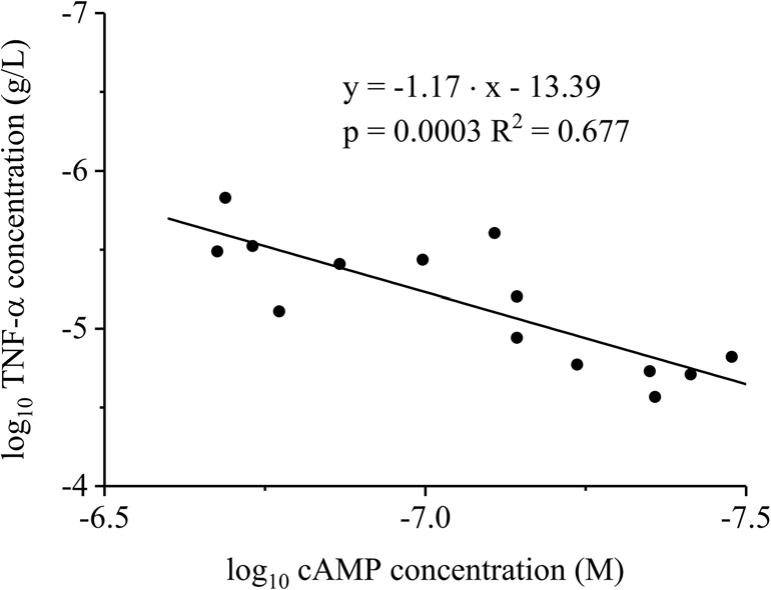
Double logarithmic plot of TNF-α plasma peak levels as a function of cAMP plasma maximal concentrations in rats with endotoxemia receiving LPS alone or simultaneously with PTX or (±)-LSF

Surprisingly, (±)-LSF administered at a dose of 40 mg kg^−1^ did not increase (*p* > 0.05) the peak cAMP level in comparison with the group receiving LPS only. The lack of statistical difference in this case may be partially explained by a small sample size. In contrast, the peak TNF-α levels following LPS administration were significantly lower (*p* < 0.05) in the presence of both compounds administered at two dose levels (Fig. 6c, d). The results of linear regression analysis indicate the occurrence of correlation between the decimal logarithms of peak cAMP and TNF-α plasma levels (Fig. 7).

Each data point was plotted using TNF-α and cAMP concentrations obtained from the same rat. The correlation between plasma levels of cAMP and those of a commonly used biomarker of inflammation—TNF-α confirms the validity of cAMP as a marker of pharmacological response following administration of non-selective PDE inhibitors.

## Discussion

PK/PD modelling is widely used in the pharmaceutical industry worldwide not only for quantitative effect-time data analysis but also for predictions of pharmacological response over time in new experimental conditions, such as different dose, dosing schedule, new routes of administration or in the case of decreased function of elimination organs. Properly selected biomarker that reflects the clinical effect is the key to success in the development of a new drug. Biomarker is defined as a measure that characterizes, in a quantitative manner, a process, which is located on the path between drug administration and effect (Danhof et al. 2005). A good biomarker should exhibit “consistent characteristics with an acceptable sensitivity and specificity representing a specific toxicity or therapeutic effect of the drug, a specific physiological response to a treatment, a pathological progression or a physiological factor” (Bai et al. 2011). As shown in this study, cAMP measured in plasma after administration of non-selective PDE inhibitors meets these criteria.

Literature data indicate that in experimental models of inflammatory diseases, PK/PD models were built using TNF-α, IFN-γ, IL-10 or NO as markers of pharmacological response (Gozzi et al. 1999; Chakraborty et al. 2005; Wyska 2010a). The major drawback of using cytokines as biomarkers to evaluate anti-inflammatory drug effects is that their concentrations in blood are highly variable between different individuals of the same species*.* The results presented in literature show a large inter-study variation in the values of cytokine levels (several times differences), despite similar experimental conditions. For example, after intraperitoneal injection of LPS at an identical dose to the same strain of mice, the reported levels of TNF-α differed several times (Gozzi et al. 1999; Wyska 2010a). This diversity may be due to the methods of blood sampling and further processing of the biological material (e.g., time and temperature of centrifugation, use of anticoagulants, sample storage conditions or the time allowed for blood clotting). A similar effect has been found in studies involving the CLP-induced model of sepsis (Otero-Antón et al. 2001; Singleton et al. 2003). cAMP as a non-protein biomarker seems to be less prone to these factors. As a result, the values of endogenous concentrations of this nucleotide observed in the present study are close to those found in the literature (Gomaa et al. 2003; Itoh et al. 2004). Another disadvantage of using cytokines as markers of pharmacological effect is that the increase in their levels is observed with a delay relative to the stimulus, which may be somewhat problematic in PK/PD modelling, as it requires using an additional tlag (lag time) parameter (Lon et al. 2012). cAMP is not burdened with this disadvantage because the increase in its levels begins immediately after administration of cAMP-elevating compounds, and it attains the peak level between 30 and 60 min thereafter.

cAMP has been frequently measured in in vitro studies, including those on pharmacodynamics of PDE inhibitors (D’Alessandro et al. 2013; Huang et al. 2015; Massimi et al. 2017). Moreover, there are several examples of measuring its concentrations in animal tissues (Kitazawa et al. 1999; Jin et al. 2004; Park et al. 2016) or plasma (Cheng et al. 1997; Tanahashi et al. 1999). However, the pharmacodynamic data obtained in these studies have not been analysed quantitatively. The only exception is the study of Jusko and coworkers, where cAMP levels in liver following acute and chronic methylprednisolone administration in adrenalectomized rats were modelled using the IDR model II (Jin et al. 2004). cAMP plasma levels have been also monitored in humans. Baseline levels of cAMP in plasma of healthy subjects obtained from different studies vary from 13 to 25 pmol mL^−1^ (Nishikimi et al. 1995; Amado et al. 1999; Cocks et al. 2000; Li and Liu 2013). Elevated levels of cAMP were observed in patients with major blunt trauma (Cocks et al. 2000), heart failure (Nishikimi et al. 1995), after thoracic epidural analgesia (Li and Liu 2013), after heart surgery under cardiopulmonary bypass, which triggers a systemic inflammatory response (Amado et al. 1999), as well as in patients treated with antiplatelet drug—ticagrelor (Li et al. 2017). Furthermore, melatonin (pineal hormone) administered orally significantly augmented cAMP plasma levels in humans 1 h following its administration (Zhdanova and Raz 1999). The extent of mean cAMP level elevation in these conditions and treatments was not higher than two times in comparison to that observed in control groups. Following intravenous infusion of PTX at a dose of 300 mg in 1 h to healthy subjects, cAMP plasma levels measured 1 h from the beginning of infusion were elevated by approximately 50% when compared to the placebo group (Kruuse et al. 2000). To our knowledge, there is a lack of information in the literature about the influence of sepsis state on cAMP levels in human plasma; thus, this issue needs further research. Due to the potential use of PDE inhibitors in the treatment of a wide range of inflammatory diseases, it would be necessary to investigate the effect of these diseases on cAMP levels in plasma before applying cAMP as a marker of pharmacological response in PK/PD modelling. Our own unpublished data show that in cecal ligation and puncture (CLP)-induced model of sepsis, which is probably the most frequently used as it closely resembles the progression and characteristics of human sepsis, cAMP serum concentrations in mice were reduced at 24 and 72 h following CLP procedure and were 26.4 (±3.5), 28.9 (±0.5), 18.0 (±0.4) and 9.3 pmol mL^−1^ (±0.5) in the control group and at 8, 24 and 72 h after CLP procedure, respectively. Therefore, when using this model of sepsis and cAMP as a biomarker, the baseline response should be described by a more complex equation.

It is well known that cAMP participates in multiple downstream pathways, but one of its most important actions is activation of the cAMP-dependent protein kinase A (PKA) I (Walsh et al. 1968). This nucleotide binds to the regulatory subunits of PKA I, leading to its dissociation into the regulatory and catalytic subunits. Subsequently, catalytic subunits of PKA I phosphorylate specific residues on multiple target proteins initiating the appropriate signalling pathways (Walsh and Van Patten 1994). cAMP-activated PKA I binds and phosphorylates cAMP-responsive transcription factors, such as cAMP-response element binding protein (CREB) (Shaywitz and Greenberg 1999), activating transcription factor-1 (ATF-1) (Rehfuss et al. 1991), nuclear factor kappa-light-chain-enhancer of activated B cells (NFκB) (Houslay et al. 2005) and nuclear receptors (Martin et al. 2009; Liu et al. 2009). This pathway is responsible for the regulation of immune response and the production of inflammatory cytokines (Jankowska et al. 2017). Moreover, elevated levels of cAMP cause down-regulation of T cell proliferation and effector functions. The mechanism of this activity is not entirely clarified, but there are some indications that it may be associated with the inducible cAMP early repressor (ICER) (Bodor et al. 1996).

Till today, two independent studies have shown that inhibition of PDE4 isoform is responsible for the elevated levels of cAMP in animal blood. Rolipram (a selective PDE4 inhibitor) but no cilostamide (a selective PDE3 inhibitor) elevated plasma cAMP concentration in dogs in a dose-dependent manner (Tanahashi et al. 1999). Similarly, CP-80,633 and rolipram, selective PDE4 inhibitors administered at the same dose of 10 mg kg^−1^ p.o., increased plasma cAMP concentrations in mice. In the same study, selective inhibitors of PDE1 (vinpocetine), PDE2 (dipyridamole), PDE3 (SKF-94,120), and PDE5 (zaprinast), given at a 10 times higher dose than that of the PDE4 inhibitors, failed to alter cAMP levels significantly (*p* < 0.05) in these tests. Moreover, a non-selective PDE inhibitor theophylline, when administered orally at a dose of 100 mg kg^−1^ to mice, increased plasma levels of cAMP up to 296.9 pmol mL^−1^ at 20 min post-dose (Cheng et al. 1997), whereas after i.p. dose of 50 mg kg^−1^ to rats with endotoxemia, it elevated concentrations of this nucleotide to 228.22 pmol mL^−1^ 0.5 h after drug administration (our own unpublished data). It is quite possible that basophiles, mast cells, neutrophils, eosinophils, monocytes and lymphocytes, which contain PDE4 and PDE7 enzymes (Torphy and Undem 1991; Smith et al. 2003), are involved in the accumulation and efflux of cAMP as a result of the treatment with non-selective as well as PDE4 and PDE7-selective PDE inhibitors (Cheng et al. 1997). Taken together, these data suggest that plasma concentrations of cAMP elevated by PDE inhibitors may be connected with an increased cAMP export from PDE4-sensitive immune cells and PDE7 inhibition may enhance this effect.

In the in vitro study conducted using human recombinant PDE4B and PDE7A, the main cAMP-hydrolysing enzymes, the IC_50_ values of IBMX as a reference compound were similar to those found in literature (Hatzelmann et al. 1995; Smith et al. 2004). We noticed a higher PDE inhibitory potency of (±)-LSF enantiomers in comparison to PTX. Moreover, both R-(-)-LSF and S-(+)-LSF demonstrated comparable IC_50_ values as PDE4B and PDE7A inhibitors. Therefore, in the pharmacodynamic models employed in this study, it was assumed that both enantiomers of LSF exhibit the same PDE inhibitory potency. Up to date, it has been believed that only R-(-)-enantiomer of (±)-LSF exhibits pharmacological activity (Nicklasson et al. 2002). For example, it has been demonstrated that R-(-)-LSF, unlike S-(+)-LSF, inhibited IL-12-induced murine T helper 1 (Th1) differentiation in a dose-dependent manner (Bright et al. 1998). In this paper, we demonstrated for the first time that both enantiomers of (±)-LSF act with a similar potency at least as PDE4B and PDE7A inhibitors. Thus, the differences observed in previous studies may arise from other mechanisms of anti-inflammatory activity of R-(-)-LSF. It has been shown that this enantiomer may act through multiple mechanisms, among which inhibition of STAT-4 (Yang et al. 2003) or phosphatidic acid generation (Bleich et al. 1996) can be mentioned as examples. However, little is known about properties of S-(+)-LSF, leaving a gap for further research. As PTX and (±)-LSF undergo metabolic interconversion in blood (Nicklasson et al. 2002; Wyska et al. 2006) and both compounds exert anti-inflammatory effects, we made an attempt to assess the nature of possible interaction that might occur. CI values calculated using CompuSyn software for PTX and R-(-)-LSF as well as for PTX and (±)-LSF oscillated around 1, indicating existence of additive (zero) interaction between both investigated compounds. This observation was taken into account during pharmacodynamic model building and finally, the additive interaction model was used in PK/PD analysis.

As mentioned above, PTX and R-(-)-LSF are prone to metabolic interconversion. The reduction of PTX to R-(-)-LSF is mediated by an enzyme independent on cytochrome P450, namely carbonyl reductase; on the other hand, the opposite reaction is catalysed by CYP1A2 (Lee and Slattery 1997). Moreover, PTX is metabolised to S-(+)-LSF. Thereby, after administration of PTX or (±)-LSF to rats, all three compounds, namely PTX and both enantiomers of (±)-LSF, occur in blood. Therefore, our PK models have some limitations. As we used a non-chiral method for determination of (±)-LSF concentrations, the parameter values obtained for (±)-LSF in fact relate to the combination of the two enantiomers co-occurring in rat plasma. The results of the previous study indicated that interconversion rates of PTX and R-(-)-LSF, as well as PTX and S-(+)-LSF, are different and that this process plays a minor role in the pharmacokinetics of both compounds (Wyska et al. 2006). Despite the simplification of pharmacokinetic models in this study, both pharmacokinetic models very well captured changes in concentrations of the tested compounds in rat plasma. The pharmacokinetic analysis confirmed the results of previous research on mice indicating the presence of non-linear pharmacokinetics of R-(-)-LSF administered alone or concomitantly with LPS (Wyska 2010a; Wyska et al. 2015). Unlike PTX, this compound is metabolized in mice principally by cytochrome P450 (Lee and Slattery 1997; Wyska 2010b); thus, the saturation of metabolism at higher doses may occur. Both PTX and (±)-LSF are rapidly eliminated from the rat body, as demonstrated by the fast decline of the terminal phase of the concentration versus time profiles. The elimination half-life (t_0.5_) of PTX equalled 16.4 min, and it was two times longer than that observed in mice (Wyska et al. 2007). In turn, the values of K_m_ or V_max_ of (±)-LSF estimated in rats (Table 2) were somehow lower than those obtained for R-(-)-LSF in mice (15.08 mg L^−1^ and 2.33 mg min^−1^ kg^−1^, respectively) (Wyska et al. 2007).

In this study, we used cAMP as a marker of pharmacological response after administration of compounds that modulate the level of this nucleotide and evaluated the in vivo potency of the investigated compounds using PK/PD modelling. As demonstrated in this paper, after administration of both PDE inhibitors, cAMP levels changed in a dose-dependent manner. The proposed PK/PD models are based on the assumption that both compounds (PTX and (±)-LSF) cause a reduction in degradation of cAMP by PDE (principally PDE4) inhibition. The first tested PK/PD model, which is the basic IDR model II (Fig. 1a) is simpler and can be successfully used in a situation where only one compound, which exhibits pharmacological activity, occurs in blood. The second model—the interaction IDR model II (Fig. 1b)—reflects a more complex situation when two active compounds are present in blood, and they undergo additive interaction. Furthermore, each compound has a different potency. This approach is based on the additive drug–drug interaction model developed by Ariens et al. (1957). The PK/PD analysis revealed that the second model (Fig. 1b) better described changes of cAMP levels over time following administration of studied compounds. It may be justified by the fact that both parent compounds and their active metabolites occur in rat blood, and they exhibit pharmacological activity as PDE inhibitors. Basic assumptions of this model are that both parent compounds and their respective active metabolites exhibit additive (zero) interaction (as it can be concluded from the results of CI analysis), and both enantiomers of (±)-LSF have the same potency as PDE inhibitors (based on the similar IC_50_ values obtained in the in vitro study). As the investigated compounds at higher concentrations fully inhibited activity of the enzymes in the in vitro test, in both compared pharmacodynamic models, I_max_ value was fixed to 1. The employed PK/PD models allowed for assessment of the PDE inhibitory potency of tested compounds in vivo. Based on the obtained results, it seems that (±)-LSF is a slightly stronger inhibitor of PDEs in rats compared to PTX, as demonstrated by approximately 20% lower IC_50_ value (Table 2). Also, the results of the in vitro test confirmed the observation that both (±)-LSF enantiomers acted stronger as hrPDE4B and hrPDE7A inhibitors compared to PTX. IC_50_ values of R-(-)-LSF and S-(+)-LSF versus PTX as hrPDE4B inhibitors were about two times lower. Moreover, IC_50_ values of PTX and (±)-LSF calculated using PK/PD model after conversion to μM are of the same order of magnitude as IC_50_ values assessed for both compounds using hrPDE4B (main cAMP hydrolysing enzyme) in the in vitro experiment and were equal 16.09 and 12.23 μM for PTX and (±)-LSF, respectively. Although enzymes used in the in vitro assay are human recombinant proteins, it has been demonstrated that PDE4 inhibitors have very similar IC_50_ values in relation to human and rat recombinant PDE4B (Bian et al. 2004). However, it must be kept in mind that PDE4 activity in rat blood leukocytes is higher in comparison to that in human cells, probably as a result of approximately 25 times higher expression of PDE4 in rat leukocytes. Anti-inflammatory potency of PTX and R-(-)-LSF has been assessed earlier using TNF-α as a marker of pharmacological response and different PK/PD models (Wyska 2010a). The results of these studies indicated the presence of dose-dependent relationship between PTX or R-(-)-LSF exposure and inhibition of TNF-α production in serum of LPS-treated mice. The value of TNF-α IC_50_ for PTX estimated in that experiment was lower than that observed for R-(-)-LSF, but the influence of S-(+)-LSF or (±)-LSF on TNF-α levels was not investigated. In this study, we evaluated the influence of PTX and (±)-LSF administration on TNF-α plasma levels in endotoxemic rats. These compounds at both doses caused a significant decrease (*p* < 0.05) in TNF-α concentrations. It may be concluded that the elevated levels of cAMP in rat plasma correspond with the decreased TNF-α levels after administration of both investigated PDE inhibitors simultaneously with LPS. Moreover, in this study, the correlation between decimal logarithms of peak TNF-α and cAMP plasma levels in endotoxemic rats was demonstrated, indicating the potential utility of cAMP as a marker of non-selective PDE inhibitors’ effect in inflammatory diseases. This observation is in line with the results of in vitro study where a correlation has been found between the decimal logarithms of cAMP accumulation potency (EC_50_) and IC_50_ of TNF-α suppressive action for a series of non-selective PDE inhibitors (Semmler et al. 1993). However, these observations are not in agreement with the results of in vitro studies using immune cells from patients suffering from Gram-negative bacterial pneumonia, where the elevated intracellular levels of cAMP observed in human leukocytes were not correlated with the suppression of TNF-α levels (Matsumoto et al. 2011).

In conclusion, the results of this study indicated that both non-selective PDE inhibitors, PTX and (±)-LSF, differ in pharmacokinetic and pharmacodynamic characteristics. The proposed PK/PD additive interaction IDR model II accurately described the concentration–effect relationship between PTX or (±)-LSF concentrations and cAMP plasma levels. The proposed PK/PD models may serve as a tool for assessing PDE inhibitory activity of new non-selective PDE or PDE4-selective inhibitors in vivo. Moreover, due to cAMP immune weakening potential, they can be used in studies on new immunomodulatory drugs, which mechanism of action is based on the elevation of cAMP levels.

## References

[CR1] Amado J, Fidalgo I, García-Unzueta M (1999). Patients with poor preoperative ejection fraction have a higher plasma response of adrenomedullin in response to open heart surgery. Acta Anaesthesiol Scand.

[CR2] Ariens EJ, Van Rossum JM, Simonis AM (1957). Affinity, intrinsic activity and drug interactions. Pharmacol Rev.

[CR3] Bai JPF, Bell R, Buckman S (2011). Translational biomarkers: from preclinical to clinical a report of 2009 AAPS/ACCP biomarker workshop. AAPS J.

[CR4] Bian H, Zhang J, Wu P (2004). Differential type 4 cAMP-specific phosphodiesterase (PDE4) expression and functional sensitivity to PDE4 inhibitors among rats, monkeys and humans. Biochem Pharmacol.

[CR5] Bleich D, Chen S, Bursten SL, Nadler JL (1996). Lisofylline, an inhibitor of unsaturated phosphatidic acid generation, ameliorates interleukin-1 beta-induced dysfunction in cultured rat islets. Endocrinology.

[CR6] Bodor J, Spetz AL, Strominger JL, Habener JF (1996). cAMP inducibility of transcriptional repressor ICER in developing and mature human T lymphocytes. Proc Natl Acad Sci U S A.

[CR7] Bright JJ, Du C, Coon M (1998). Prevention of experimental allergic encephalomyelitis via inhibition of IL-12 signaling and IL-12-mediated Th1 differentiation: an effect of the novel anti-inflammatory drug lisofylline. J Immunol.

[CR8] Chakraborty A, Yeung S, Pyszczynski NA, Jusko WJ (2005). Pharmacodynamic interactions between recombinant mouse interleukin-10 and prednisolone using a mouse endotoxemia model. J Pharm Sci.

[CR9] Cheng JB, Watson JW, Pazoles CJ (1997). The phosphodiesterase type 4 (PDE4) inhibitor CP-80,633 elevates plasma cyclic AMP levels and decreases tumor necrosis factor-alpha (TNFalpha) production in mice: effect of adrenalectomy. J Pharmacol Exp Ther.

[CR10] Chou T-C (2006). Theoretical basis, experimental design, and computerized simulation of synergism and antagonism in drug combination studies. Pharmacol Rev.

[CR11] Cocks RA, Rainer TH, Chan TY (2000). Increased plasma free cyclic-AMP levels following major trauma and their relevance to the immune response. Resuscitation.

[CR12] D’Alessandro L, Petrini M, Ferrante M (2013). Cyclic nucleotide phosphodiesterase activity in stem cells of human periodontal ligament (PDL-MSCs) before and after osteogenic induction. Oral Surg Oral Med Oral Pathol Oral Radiol.

[CR13] Danhof M, Alvan G, Dahl SG (2005). Mechanism-based pharmacokinetic–pharmacodynamic modeling—a new classification of biomarkers. Pharm Res.

[CR14] European Medicines Agency (EMA) (2011) Guideline on bioanalytical method validation. http://www.ema.europa.eu/docs/en_GB/document_library/Scientific_guideline/2011/08/WC500109686.pdf. Accessed 30 May 2016

[CR15] Ferguson NR, Galley HF, Webster NR (1999). T helper cell subset ratios in patients with severe sepsis. Intensive Care Med.

[CR16] Francis SH, Blount MA, Corbin JD (2011). Mammalian cyclic nucleotide phosphodiesterases: molecular mechanisms and physiological functions. Physiol Rev.

[CR17] Gomaa A, Hashem T, Mohamed M, Ashry E (2003). *Matricaria chamomilla* extract inhibits both development of morphine dependence and expression of abstinence syndrome in rats. J Pharmacol Sci.

[CR18] González-García C, Bravo B, Ballester A (2013). Comparative assessment of PDE 4 and 7 inhibitors as therapeutic agents in experimental autoimmune encephalomyelitis. Br J Pharmacol.

[CR19] Gozzi P, Pahlman I, Palmer L (1999). Pharmacokinetic-pharmacodynamic modeling of the immunomodulating agent susalimod and experimentally induced tumor necrosis factor-alpha levels in the mouse. J Pharmacol Exp Ther.

[CR20] Harada H, Ishizaka A, Yonemaru M (1989). The effects of aminophylline and pentoxifylline on multiple organ damage after *Escherichia coli* sepsis. Am Rev Respir Dis.

[CR21] Hatzelmann A, Tenor H, Schudt C (1995). Differential effects of non-selective and selective phosphodiesterase inhibitors on human eosinophil functions. Br J Pharmacol.

[CR22] Houslay MD, Schafer P, Zhang KYJ (2005). Keynote review: phosphodiesterase-4 as a therapeutic target. Drug Discov Today.

[CR23] Huang Y, Zheng L, Yang H (2015). Measuring the dynamics of cyclic adenosine monophosphate level in living cells induced by low-level laser irradiation using bioluminescence resonance energy transfer. J Biomed Opt.

[CR24] Itoh T, Nagaya N, Fujii T (2004). A combination of oral sildenafil and beraprost ameliorates pulmonary hypertension in rats. Am J Respir Crit Care Med.

[CR25] Jankowska A, Świerczek A, Chłoń-Rzepa G et al (2017) PDE7-selective and dual inhibitors: advances in chemical and biological research. Curr Med Chem. doi:10.2174/0929867324666170116125159 10.2174/0929867324666170116125159 28093982

[CR26] Jin JY, DuBois DC, Almon RR, Jusko WJ (2004). Receptor/gene-mediated pharmacodynamic effects of methylprednisolone on phosphoenolpyruvate carboxykinase regulation in rat liver. J Pharmacol Exp Ther.

[CR27] Kitazawa T, Takaoka K, Taneike T (1999). Mechanisms of 5-hydroxytryptamine-induced inhibition in the porcine myometrium. J Auton Pharmacol.

[CR28] Kruuse C, Jacobsen TB, Thomsen LL (2000). Effects of the non-selective phosphodiesterase inhibitor pentoxifylline on regional cerebral blood flow and large arteries in healthy subjects. Eur J Neurol.

[CR29] Kunz M, Ibrahim SM (2009). Cytokines and cytokine profiles in human autoimmune diseases and animal models of autoimmunity. Mediat Inflamm.

[CR30] László I, Trásy D, Molnár Z, Fazakas J (2015). Sepsis: from pathophysiology to individualized patient care. J Immunol Res.

[CR31] Lee SH, Slattery JT (1997). Cytochrome P450 isozymes involved in lisofylline metabolism to pentoxifylline in human liver microsomes. Drug Metab Dispos.

[CR32] Li QS, Liu FQ (2013). Effects of thoracic epidural analgesia on plasma cAMP and cGMP levels in patients with heart failure. J Cardiothorac Surg.

[CR33] Li X, Wang Q, Xue Y (2017). Ticagrelor compared with clopidogrel increased adenosine and cyclic adenosine monophosphate plasma concentration in acute coronary syndrome patients. Basic Clin Pharmacol Toxicol.

[CR34] Liu N-C, Lin W-J, Yu I-C (2009). Activation of TR4 orphan nuclear receptor gene promoter by cAMP/PKA and C/EBP signaling. Endocrine.

[CR35] Lon H-K, Liu D, Jusko WJ (2012). Pharmacokinetic/pharmacodynamic modeling in inflammation. Crit Rev Biomed Eng.

[CR36] Martin LJ, Boucher N, El-Asmar B, Tremblay JJ (2009). cAMP-induced expression of the orphan nuclear receptor Nur77 in MA-10 Leydig cells involves a CaMKI pathway. J Androl.

[CR37] Massimi M, Cardarelli S, Galli F (2017). Increase of intracellular cyclic AMP by PDE4 inhibitors affects HepG2 cell cycle progression and survival. J Cell Biochem.

[CR38] Matsumoto T, Hayamizu K, Marubayashi S (2011). Relationship between the cAMP levels in leukocytes and the cytokine balance in patients surviving gram negative bacterial pneumonia. J Clin Biochem Nutr.

[CR39] Nicklasson M, Björkman S, Roth B (2002). Stereoselective metabolism of pentoxifylline in vitro and in vivo in humans. Chirality.

[CR40] Nishikimi T, Saito Y, Kitamura K (1995). Increased plasma levels of adrenomedullin in patients with heart failure. J Am Coll Cardiol.

[CR41] Noack M, Miossec P (2014). Th17 and regulatory T cell balance in autoimmune and inflammatory diseases. Autoimmun Rev.

[CR42] Otero-Antón E, González-Quintela A, López-Soto A (2001). Cecal ligation and puncture as a model of sepsis in the rat: influence of the puncture size on mortality, bacteremia, endotoxemia and tumor necrosis factor alpha levels. Eur Surg Res.

[CR43] Park JH, Kim SE, Jin JJ (2016). Pentoxifylline alleviates perinatal hypoxic-ischemia-induced short-term memory impairment by suppressing apoptosis in the hippocampus of rat pups. Int Neurourol J.

[CR44] Raker VK, Becker C, Steinbrink K (2016). The cAMP pathway as therapeutic target in autoimmune and inflammatory diseases. Front Immunol.

[CR45] Rehfuss RP, Walton KM, Loriaux MM, Goodman RH (1991). The cAMP-regulated enhancer-binding protein ATF-1 activates transcription in response to cAMP-dependent protein kinase A. J Biol Chem.

[CR46] Rice GC, Brown PA, Nelson RJ (1994). Protection from endotoxic shock in mice by pharmacologic inhibition of phosphatidic acid. Proc Natl Acad Sci U S A.

[CR47] Selmi C (2011). Autoimmunity in 2010. Autoimmun Rev.

[CR48] Semmler J, Gebert U, Eisenhut T (1993). Xanthine derivatives: comparison between suppression of tumour necrosis factor-alpha production and inhibition of cAMP phosphodiesterase activity. Immunology.

[CR49] Shaywitz AJ, Greenberg ME (1999). CREB: a stimulus-induced transcription factor activated by a diverse array of extracellular signals. Annu Rev Biochem.

[CR50] Singleton KD, Wischmeyer PE, Wischmeyer AP (2003). Distance of cecum ligated influences mortality, tumor necrosis factor-alpha and interleukin-6 expression following cecal ligation and puncture in the rat. Eur Surg Res.

[CR51] Smith SJ, Brookes-Fazakerley S, Donnelly LE (2003). Ubiquitous expression of phosphodiesterase 7A in human proinflammatory and immune cells. Am J Physiol Lung Cell Mol Physiol.

[CR52] Smith SJ, Cieslinski LB, Newton R (2004). Discovery of BRL 50481 [3-(N,N-dimethylsulfonamido)-4-methyl-nitrobenzene], a selective inhibitor of phosphodiesterase 7: in vitro studies in human monocytes, lung macrophages, and CD8+ T-lymphocytes. Mol Pharmacol.

[CR53] Tanahashi M, Hara S, Yoshida M (1999). Effects of rolipram and cilostamide on renal functions and cyclic AMP release in anesthetized dogs 1. J Pharmacol Exp Ther.

[CR54] Torphy TJ, Undem BJ (1991). New drugs review phosphodiesterase inhibitors: new opportunities for the treatment of asthma. Thorax.

[CR55] Walsh DA, Van Patten SM (1994). Multiple pathway signal transduction by the cAMP-dependent protein kinase. FASEB J.

[CR56] Walsh DA, Perkins JP, Krebs EG (1968). An adenosine 3′,5′-monophosphate-dependant protein kinase from rabbit skeletal muscle. J Biol Chem.

[CR57] Wyska E (2010). Pharmacokinetic-pharmacodynamic modeling of methylxanthine derivatives in mice challenged with high-dose lipopolysaccharide. Pharmacology.

[CR58] Wyska E (2010). Pharmacokinetic interaction between verapamil and methylxanthine derivatives in mice. Drug Metab Lett.

[CR59] Wyska E, Pekala E, Szymura-Oleksiak J (2006). Interconversion and tissue distribution of pentoxifylline and lisofylline in mice. Chirality.

[CR60] Wyska E, Świerczek A, Pociecha K, Przejczowska-Pomierny K (2015). Physiologically based modeling of lisofylline pharmacokinetics following intravenous administration in mice. Eur J Drug Metab Pharmacokinet.

[CR61] Wyska E, Szymura-Oleksiak J, Pekala E (2007). Pharmacokinetic modelling of pentoxifylline and lisofylline after oral and intravenous administration in mice. J Pharm Pharmacol.

[CR62] Yang Z-D, Chen M, Wu R (2002). The anti-inflammatory compound lisofylline prevents type I diabetes in non-obese diabetic mice. Diabetologia.

[CR63] Yang Z, Chen M, Fialkow LB (2003). Inhibition of STAT4 activation by lisofylline is associated with the protection of autoimmune diabetes. Ann N Y Acad Sci.

[CR64] Yang Z, Chen M, Nadler JL (2005). Lisofylline: a potential lead for the treatment of diabetes. Biochem Pharmacol.

[CR65] Zhdanova IV, Raz DJ (1999). Effects of melatonin ingestion on cAMP and cGMP levels in human plasma. J Endocrinol.

